# Soil application of effective microorganisms and nitrogen alleviates salt stress in hot pepper (*Capsicum annum* L.) plants

**DOI:** 10.3389/fpls.2022.1079260

**Published:** 2023-01-18

**Authors:** Abdelsattar Abdelkhalik, Taia A. Abd El-Mageed, Ibrahim A. A. Mohamed, Wael M. Semida, Omar A. A. I. Al-Elwany, Ibrahim M. Ibrahim, Khaulood A. Hemida, Mohamed T. El-Saadony, Synan F. AbuQamar, Khaled A. El-Tarabily, Mohammed A. H. Gyushi

**Affiliations:** ^1^ Horticulture Department, Faculty of Agriculture, Fayoum University, Fayoum, Egypt; ^2^ Soil and Water Department, Faculty of Agriculture, Fayoum University, Fayoum, Egypt; ^3^ Botany Department, Faculty of Agriculture, Fayoum University, Fayoum, Egypt; ^4^ Department of Agricultural Microbiology, Faculty of Agriculture, Fayoum University, Fayoum, Egypt; ^5^ Botany Department, Faculty of Science, Fayoum University, Fayoum, Egypt; ^6^ Department of Agricultural Microbiology, Faculty of Agriculture, Zagazig University, Zagazig, Egypt; ^7^ Department of Biology, College of Science, United Arab Emirates University, Al Ain, United Arab Emirates; ^8^ Khalifa Center for Genetic Engineering and Biotechnology, United Arab Emirates University, Al Ain, United Arab Emirates; ^9^ Harry Butler Institute, Murdoch University, Murdoch, WA, Australia

**Keywords:** biostimulants, *Capsicum annuum*, growth, osmotic adjustment, salinity stress, yield

## Abstract

The application of effective microorganisms (EMs) and/or nitrogen (N) have a stimulating effect on plants against abiotic stress conditions. The aim of the present study was to determine the impact of the co-application of EMs and N on growth, physio-biochemical attributes, anatomical structures, nutrients acquisition, capsaicin, protein, and osmoprotectant contents, as well as the antioxidative defense system of hot pepper (*Capsicum annum* L.) plants. In the field trials, EMs were not applied (EMs^-^) or applied (EMs^+^) along with three N rates of 120, 150, and 180 kg unit N ha^-1^ (designated as N_120_, N_150_, and N_180_, respectively) to hot pepper plants grown in saline soils (9.6 dS m^-1^). The application of EMs and/or high N levels attenuated the salt-induced damages to hot pepper growth and yield. The application of EMs^+^ with either N_150_ or N_180_ increased the number, average weight and yield of fruits by 14.4 or 17.0%, 20.8 or 20.8% and 28.4 or 27.5%, respectively, compared to hot pepper plants treated with the recommended dose (EMs^-^ × N_150_). When EMs^+^ was individually applied or combined with either N_150_ or N_180_, increased accumulation of capsaicin were observed by 16.7 or 20.8%, protein by 12.5 or 16.7%, proline by 19.0 or 14.3%, and total soluble sugars by 3.7 or 7.4%, respectively, in comparison with those treated with the integrative EMs^-^ × N_150_. In addition, the non-enzymatic contents (ascorbate, and glutathione) and enzymatic activities (catalase, superoxide dismutase, and glutathione reductase) of the antioxidant defense systems significantly increased in hot pepper plants treated with EMs^+^ alone or combined with N_150_ or N_180_ under salt stress conditions. Higher accumulation of nutrients (N, P, K^+^, and Ca^2+^) along with reduced Na^+^ acquisition was also evidenced in response to EMs^+^ or/and high N levels. Most anatomical features of stems and leaves recovered in hot pepper plants grown in saline soils and supplied with EMs^+^ and N. The application of EMs and N is undoubtedly opening new sustainable approaches toward enhancing abiotic stress tolerance in crops (e.g. hot pepper).

## 1 Introduction

Hot pepper (*Capsicum annuum* L.) is a widely cultivated vegetable crop and is generally consumed due to its nutritive content, biologically active and antioxidant compounds, and pungency (Lahbib et al., 2017). In Egypt, hot pepper are mostly grown in saline non-fertile soil, resulting in fruit yield losses (Abd El-Mageed et al., 2020).

Soil salinity is one of the main factors responsible for crop yield losses in many regions of the world (Abid et al., 2020). Cultivated and irrigated salt-affected soils are approximately 20-30% worldwide; and the salinized areas are expected to double by 2050 due to the high evapotranspiration, low rainfall, poor agricultural sustainable practices and improper irrigation and drainage (Machado and Serralheiro, 2017; Kaya et al., 2020a; Semida et al., 2021a). Salinity induced complex mechanisms that modulate various physio-biochemical and molecular pathways of plants (Kamran et al., 2020). Initially, soil salinity decreases the soil osmotic potential, resulting in osmotic stress that reduces root water content and nutrient uptake (Zhonghua et al., 2011; Gupta and Huang, 2014). Ionic stress is caused by an excess accumulation of hazardous ions (Na^+^ and Cl^−^) in plant tissues (Arif et al., 2020).

Salinity stress mediates the overproduction of reactive oxygen species (ROS) that provokes oxidative damage to the plasma membrane and organelles (i.e. mitochondria and chloroplast), protein biosynthesis, disruption of ion homeostasis, photosynthesis, and downregulation of antioxidant activities in the plant cell (Shelke et al., 2019; Abd El-Mageed et al., 2020). Excessive Na^+^ and Cl^−^ buildup cause cytosolic K^+^ and Ca^2+^ efflux, resulting in a cellular homeostasis imbalance, and nutritional deficiency (Ahanger et al., 2017). High salt concentrations in the soil exacerbated decreases in leaf water content, membrane integrity, photosynthetic pigment biosynthesis, and osmoprotectant levels (Ahmad et al., 2016; Kaya and Ashraf, 2020). The oxidative stress, caused by salinity stress, damages the reaction centers of photosystem II (PSII), resulting in a decrease in PSII activity (Akhter et al., 2021).

To cope with the salinity-induced growth reduction, plants adapted indigenously protective mechanisms *via* up-regulating antioxidant activity, osmoregulatory substances, and other secondary metabolites (Semida et al., 2020; Rady et al., 2021a). Moreover, the accumulation of selective mineral ions in plants exhibited salinity stress tolerance (Ahanger et al., 2019). Therefore, convenient management approaches are required to ameliorate the harmful impacts of salinity on crop performance. Proper mineral nutrition and/or supplementation of effective microorganisms (EMs) may be suitable ways to enhance plant tolerance potential to salt stress (Khan et al., 2017; Sikder et al., 2020).

Nitrogen (N) is a critical plant component and one of the most important yield-limiting elements in crops (Guo et al., 2019) since N is required for amino acids synthesis, which is a major component of chlorophyll, nucleotides, and proteins (Nunes-Nesi et al., 2010). The availability of N regulates hormone synthesis, secondary metabolites, osmolytes, and antioxidant metabolism (Ahanger et al., 2019). The plant’s N content decreases in response to salinity for a variety of reasons, including decreased N uptake, the antagonism between Cl^−^ and ${\text{NO}}_{3}^{-}$ , and Na^+^ and ${\text{NH}}_{4}^{+}$ , the absence of ATP compounds that are necessary to activate the transport of ${\text{NO}}_{3}^{-}$ , and a change in the enzymatic activity involved in ${\text{NH}}_{4}^{+}$ and ${\text{NO}}_{3}^{-}$ -metabolism (Ashraf et al., 2018; Piñero et al., 2019). In salt-affected soils, an insufficient N status is frequently the growth-limiting issue. As a result, whether the crop is salt-stressed or not, adding N usually boosts plant performance (growth and yield) (Semiz et al., 2014). N supplementation has been found to enhance the resistance to abiotic stress (Ahanger et al., 2019; Song et al., 2019; Li et al., 2020).

Under saline conditions, N-supplemented plants resulted in increased stomatal conductance, photosynthetic rate, CO_2_ fixation, chlorophyll fluorescence, and up-regulated the plant’s defense compounds, including antioxidants, and osmoprotectants (Ahanger et al., 2019; Sikder et al., 2020). Under salinity stress, increased N application up to 400 mg pot^-1^ markedly increased chlorophyll fluorescence parameters, photosynthetic rate, dry biomass, and grain yield of two oat genotypes (Song et al., 2019). High N supplementation improved water status, membrane integrity, chlorophyll fluorescence, SPAD chlorophyll index, and grain yield in three rice varieties under water deficits conditions (Abdou et al., 2021).

Recently, plant growth-promoting microorganisms have been widely used to boost nutrients acquisition, productivity, and growth of many crops (El-Tarabily et al., 2021; Orozco-Mosqueda et al., 2022). As ecofriendly biofertilizers, effective microorganisms (EMs) have remarkable capabilities in combating salinity stress, improving plant growth and enhancing crop productivity; thus, contributing to food security (Naik et al., 2020). EMs are made up of a wide variety of naturally coexisting useful microorganisms, including lactic acid bacteria, photosynthetic bacteria, actinobacteria, fungi, and yeasts (Hu and Qi, 2013; Naik et al., 2020). EMs have a stimulating effect on plants because they function as soil activators, and biostimulants (Iriti et al., 2019; Alkaabi et al., 2022). EMs improve soil physicochemical properties, organic matter decomposition, soil fertility, and nutrient solubility, as well as aid in the control and suppression of soil pathogens and pests, reducing the need for synthetic agrochemicals and increasing profitability and sustainability (Hu and Qi, 2013; El-Tarabily et al., 2021; Alwahshi et al., 2022).

Similar to biostimulants, the EMs mixtures can produce biologically active molecules such as lactic acid, vitamins, amino acids, esters, sugars, enzymes, and hormones (Iriti et al., 2019; Abd El-Mageed et al., 2020). Such beneficial substances stimulate root development, photosynthesis, protein activity, and yield of crops (Abd El-Mageed et al., 2022). Furthermore, evidence has been found in some studies that EMs can reduce the negative effects of soil salinity and promote salt tolerance (Talaat, 2015; Numan et al., 2018; Naik et al., 2020; Fadiji et al., 2022; Orozco-Mosqueda et al., 2022). Exogenous application of EMs increased plant water status, nutrients acquisition, photosynthetic pigments, and osmoprotectant accumulation as well as elevated the antioxidant capacity, a crucial mechanism for reducing oxidative damage (Talaat, 2014; Abd El-Mageed et al., 2022). The macronutrient contents, the efficiency of the photosystem II (PSII), and the seed yield of bean plants were all improved when the soil was supplemented with EMs (Iriti et al., 2019).

Nonetheless, there is no yet information regarding the integrative influence of N levels and EMs on the growth and productivity of hot pepper, as well as the mechanisms of salt stress mitigation. The hypothesis in the current study was that the soil application of EM and N could stimulate the defense system of the hot pepper plant to alleviate the effect of salt stress. Therefore, this study aimed to investigate the changes in water status, membrane stability, photosynthetic efficiency of the PSII, nutrients content, protein, capsaicin content, osmoprotectants, antioxidants, growth, and yield components under the influence of different N levels and/or EMs application in hot pepper grown under saline soil conditions.

## 2 Materials and methods

### 2.1 Site description and agronomic details

Open field trials were performed during two growing seasons; the spring season (SS) and the autumn season (AS) of 2020 in the horticultural experimental station of Fayoum University, Fayoum, Egypt (29°17’38”N 30°54’55”E). The climatic conditions in the local area are arid based on the aridity index classification (Ponce et al., 2000). The soil analysis in **Table 1** indicates that the soil is strongly saline (9.6 dS m^-1^), and slightly alkaline (pH 7.76). The soil has a coarse (sandy loam) texture and is moderately deep, and is classified as Typic Torripsamments, siliceous, hyperthermic (Soil Survey Staff USDA, 2014).

Seeds of hot pepper (cv. Omega F1) were sown on 25 January and 25 July 2020, in 209-cell polystyrene trays filled with a mixed substrate of peat moss and vermiculite (1:1) and the trays were then placed in the greenhouse. Transplantation took place on 10 March and 5 September 2020 (when seedlings form four true leaves) in beds of 10 m in length and one-meter width in an open field. The seedlings were transplanted 0.50 m apart, with one row of plants per bed. Each plot area included three beds. The incorporation of nutrients during the entire growth period of the hot pepper crop were applied at the recommended rates in the area; including 80 kg P ha^-1^, and 170 kg K ha^-1^. Proper agronomic management and pest control for hot pepper crop was applied following the bulletin of the Egyptian agricultural research center.

### 2.2 Experimental design and application of EMs and N levels

Experiments were carried out in a split-plot in a randomized complete block design (RCBD) with two factors; EMs were allocated the main plots, and different N levels were allocated in the subplots. Three N levels (N_120_ with 120 kg N unit ha^-1^, N_150_ with 150 kg N unit ha^-1^, and N_180_ with 180 kg N unit ha^-1^) were applied as ammonium nitrate (NH_4_NO_3_) form by fertigation from the third week after transplantation. Thereafter, each N level was divided into two weekly doses during the hot pepper growing season as follows; 15, 25, 50, and 10% of the N quantity were applied during the vegetative growth, early flowering and bearing, harvesting, and late harvest stages, respectively (Condés, 2017). The total quantity of N applied as N_120_, N_150_, and N_180_ were 360 kg ha^-1^, 455 kg ha^-1^, and 545 kg ha^-1^ of ammonium nitrate. According to the bulletin of the Egyptian Research Center, N_150_ is the recommended level for hot pepper production.

The EMs were not applied (EMs^-^) or applied (EMs^+^) at three different times after 15, 35, and 55 days of transplantation as a soil drench (simultaneously with irrigation). The EMs were used in a formulation that included five types of beneficial microorganisms, including photosynthetic bacteria (*Rhodobacter sphaeroides* and *Rhodopseudomonas palustris*), lactic acid bacteria (*Lactobacillus casei*, *Lactobacillus plantarum*, and *Streptococcus lactis*), yeast (*Candida utilis* and *Saccharomyces cerevisiae*), fermenting fungi (*Penicillium* sp., *Aspergillus oryzae*, and *Mucor hiemalis*), and actinobacteria (*Streptomyces griseus* and *Streptomyces albus*). The photosynthetic bacteria were cultivated on Luria-Bertania broth medim (LB; Lab M Limited, Lancashire, UK)), pH 7.0 at 30°C for 72 h in a shaking incubator at 200 rpm (Kang et al., 2015). The lactic acid bacteria were cultivated on MRS broth medium (Lab M Limited), pH 6.2 at 37°C for 48 h in anaerobic incubator (De Man et al., 1960). The yeasts were cultivated on YPD growth medium (Lab M Limited), pH 5.5-5.8 at 30°C for 72 h in a shaking incubator at 150 rpm (Sherman et al., 1986).

The fungi used in this study were cultivated on Czapek broth medium (Lab M Limited), pH 7.3 ± 0.2 at 28°C for 7 days in a shaking incubator at 150 rpm (Khan et al., 2015). The actinobacteria were cultivated on tryptic soy broth medium (TSB) (Lab M Limited), pH 7.3 ± 0.2 at 30°C for 7 days in a shaking incubator at 200 rpm (Nonthakaew et al., 2022). The mixture was prepared in equal proportions as an EM stock solution at the Ministry of Agriculture and Land Reclamation, Giza, Egypt, and was diluted to 1:1000 (EM: water, v/v) with a final concentration of microorganisms of 1.0×10^8^ – 2.0×10^8^ colony forming units (CFU) ml^−1^ at the application time. Each plant beside the root was then inoculated with 100 ml of suspension of each application.

### 2.3 Plant growth and fruit yield

Six representative plants from each plot area were sampled at 80 days after transplantation, and the growth parameters were determined. Plant height and number of leaves and branches plant^-1^ were recorded. Plant leaf area was assessed by washing the leaf surface after that ten discs were taken and dried at 75°C in the air-forced oven for 24 h and the disc’s dry weight was determined (DDW; g). The leaf area was determined as follows:


$${\text{Plant leaf area (dm}}^{2})=(\text{LDW}/\text{DDW})\times \text{DA}$$

Where, LDW denotes the leaf dry weight; DDW denotes the disc’s dry weight; and DA denotes the disc’s area. The plant leaves and branches were dried to constant weight in the oven at 75°C, after which the dry weight for each part and the total dry weight (called shoots) were determined. Ten plants from each experimental plot were harvested to determine the number of fruits plant^-1^, average fruit weight, and total fruit yield.

### 2.4 Assessment of membrane integrity, tissue water status, and photosynthetic capacity

Relative water content (RWC, %) and membrane stability index (MSI, %) of leaf were assessed as described by Abdelkhalik et al. (2019). Leaf MSI was determined with 0.2 g of fully expanded leaves as follows;


$$\text{MSI}\;(\%)=\left[1-\left(\frac{\text{EC}1}{\text{EC}2}\right)\right]\times 100$$

The leaves samples were inserted into a tube with 10 ml of distilled water and incubated in the water bath for 30 min at 40°C, and the solution’s electrical conductivity (EC) was measured (EC1). After heating the samples for 10 min at 100°C, the solution EC was measured (EC2).

Leaf RWC was assessed with 10 discs taken from fully expanded leaves in each plot area and calculated after obtaining the disc’s fresh (FW), saturated (SW), and dry (DW) weight as follows;


$$\text{RWC (\%)}=\frac{\left(\text{FW}-\text{DW}\right)}{\left(\text{SW}-\text{DW}\right)}\times 100$$

The photosynthetic efficiency of the PSII was determined as maximal quantum yield (*F_v_/F_m_*) and calculated with the formula: (*F_m_*-*F_0_*)/*F_m_* (Maxwell and Johnson, 2000), and the index of photosynthetic performance (PI_abs_) as described by Clark et al. (2000). The readings were taken on a sunny day between 9:00-11:00 AM from fully expanded one leaf plant^-1^ by a fluorometer (Hansatech Instruments Ltd., Kings Lynn, UK). Before conducting the measurements, the leaves were kept for 20 min for dark adaptation with light-blocking clips (Hansatech Instruments Ltd.). The relative chlorophyll content (SPAD value) was measured in fully developed leaves using a SPAD-502 chlorophyll meter (Minolta, Osaka, Japan).

### 2.5 Determination of total capsaicin, total protein, total soluble sugars (TSS) and free proline

Hot pepper total protein was determined in the leaves using Folin phenol as a reagent following the methodology of Lowry et al. (1951). Free proline assessment was obtained according to the Bates et al. (1973) method. Hot pepper plant tissues were homogenized in 10 ml of C_7_H_6_O_6_S (3%) and filtered. Two ml of the filtrate was mixed with 2 ml of CH3COOH + 2 ml of C_9_H_6_O_4_ reagent. Simultaneously, the samples were heated for 60 min at 100°C with the caps on. The resulting solution was harvested and strenuously mixed in a test tube with 4 ml toluene for 15-20 sec. Toluene-containing chromophore was withdrawn from the aqueous phase and left to ambient temperature. At 520 nm, absorbance was measured using toluene as a blank. Using the calibration curve, the proline content of the samples (mg g^-1^ fresh weight) was quantified.

TSS were quantified using ethanol (80%) extract from fresh hot pepper leaves (Irigoyen et al., 1992). The alcoholic extract (1 ml) was mixed with 3 ml of anthrone reagent [freshly prepared as 150 mg C_14_H_10_O plus 100 ml of 72% (v/v) H_2_SO_4_] and then placed in a boiling water bath 10 min, followed by cooling. The reading was read with a spectrophotometer at 625 nm. Total capsaicin was extracted and determined from the dry fruits following the methodology reported by Trejo-González and Tamirano (1973).

### 2.6 Extraction and quantification of antioxidants

For the extraction of glutathione reductase (GR), ascorbate peroxidase (APX), catalase (CAT), and superoxide dismutase (SOD), fresh samples of 500 mg were homogenized with a pre-chilled pestle and mortar in ice-cold 0.1 M phosphate buffer (pH=7.5) containing 0.5 mM EDTA. Each resulting mixture was transmitted to centrifuge tubes and centrifuged at 15000 x *g*, at 4°C for 15 min in a Beckman refrigerated centrifuge. The supernatant was used to quantify the enzyme’s activity (Bradford, 1976). The SOD activity was calculated by measuring the enzyme’s decrease in the absorbance of the superoxide-nitro blue tetrazolium complex (Kono, 1978). The absorbance was measured at 560 nm. The activity of CAT was assessed as described by Aebi (1984). The amount of H_2_O_2_ decomposed was used to calculate the enzyme activity. The reaction began with the addition of H_2_O_2_, and the reduction in the absorbance was measured at 240 nm for 1 min. The APX activity was determined following the criteria illustrated by Yoshimura et al. (2000). The GR activity was measured by measuring the increase in the absorbance when the oxidized glutathione (GSSG) and dithionitrobenzoic acid (DTNB) were present (Sairam et al., 2002).

Glutathione (GSH) was estimated using Ellman’s reagent as described by Anderson (1985). Under cold conditions, GSH was extracted with 500 mg of hot pepper tissues in 1% picric acid (w/v). At 412 nm, the absorbance was measured with a spectrophotometer. The content of ascorbate (AsA) was estimated using the Folin phenol reagent as outlined by Jagota and Dani (1982). About 500 mg of plant tissues were ground in oxalic acid and centrifuged for 5 min at 15000 x *g*, at 4°C for 15 min. After diluting 0.2-0.5 ml supernatant to 2 ml with distilled water, 0.2 ml of diluted Folin reagent was added to the extract. The tubes were shaken vigorously and the absorbance was mesured after 10 min at 760 nm.

### 2.7 Determination of nutrient contents

The determination of nutrients concentration in the hot pepper tissues were performed in leaves, which were dried and milled before chemical analysis. The leaf samples were digested using a mixture of HNO_3_ and HClO_4_ (at 3:1, v/v), and the digest solution was used for the quantification of the nutrients. The N content in hot pepper leaves was assessed according to the procedures of AOAC (2000) with a micro-Kjeldahl device (Ningbo Medical Instruments Co., Ningbo, China). The blue color method (Jackson, 1973) was used for the P assessment. Using flame photometer apparatus (Perkin-Elmer Model 52-A, Glenbrook, Stamford, CT, USA) the K^+^, Ca^2+^ and Na^+^ contents were quantified as outlined in the methods of Page et al. (1982).

### 2.8 Leaf and stem anatomical structures

The stem and leaf samples (the sixth top internode from the stem with its leaf) were taken at 80 days after transplantation for anatomical observations. The samples of leaves and stems were fixed in a formalin-acetic acid-alcohol (FAA) solution for 72 h to kill and fix the tissue sample according to Mohamed et al. (2020). The following steps of clearance, dehydration, embedding in paraffin wax, and cutting of the cross-sections by a rotary microtome (Zhejiang Jinhua Kedi Co., Ltd. China) were performed (Mohamed et al., 2020). Thereafter, the sections were stained with a crystal violet erythrosine combination and decolored sequentially by placing into 50%, 70%, and 90% gradient alcohols for 5–10 sec, then carbol xylene, and inserted in Canada balsam (Sass, 1958). The sections of stems and leaves were observed using an optical microscope instrument (AxioPlan, Zeiss, Jena, Germany) connected with a digital camera (Nikon DS-U3, Tokyo, Japan). Furthermore, image analysis was performed using the CaseViewer 2.3 program (3DHISTECH Ltd., Budapest, Hungary).

### 2.9 Statistical analyses

The analysis of variance (ANOVA) as well as the homogeneity of error variance for all features were performed. The statistical analysis for all data was carried out based on the split-plot in CRBD with Genstat software. The collected data from the two planting seasons were combined since both growing seasons behaved similarly. Differences between the single effects of growing seasons, EMs and N levels, and the interaction effect of EMs **×** N levels were compared using Fisher’s least-significant difference (LSD) test at P ≤ 0.05.

## 3 Results

### 3.1 Hot pepper growth, and yield-related parameters

First, we determined the growth characteristics of hot pepper, including plant height, number of leaves and branches, shoot dry weight, and leaf area in response to the growing seasons, EMs, N levels, and EMs × N interaction (**Table 2**). Hot pepper plants transplanted in spring recorded greater growth trait values than those transplanted in autumn, despite having the same number of branches plant^-1^.

The EMs supplemented plants grown in saline soil (EMs^+^) enhanced plant height, number of leaves, number of branches, leaf area, and shoot dry weight by an increase of 9.5, 33.3, 11.0, 24.7, and 22.3%, respectively, in comparison with non-EMs-treated plants grown under the same conditions (EMs^-^). In addition, the fertilization with N_150_ and N_180_ increased growth parameters relative to plants that received a lower N dose (N_120_). The co-application of EMs^+^ and N showed a stimulatory effect on hot pepper growth under saline soil conditions. The combined EMs^+^ × N_150_ or EMs^+^ × N_180_ increased plant height (by 10.6 or 12.9%, respectively), leaf number (by 41.9 or 30.8%, respectively), number of branches (by 2.5 or 12.5%, respectively), leaf area (by 18.1 or 21.2%, respectively), and shoot dry weight (by 24.2 or 20.6%, respectively), compared to those received the recommended N dose and non-amended with EMs (EMs^-^ × N_150_) (**Table 2**).

Hot pepper plants cultivated in spring recorded higher fruit yield than those cultivated in autumn; while both seasons recorded similar fruit number and average fruit weight (**Table 2**). Under high salt stress conditions, plants not treated with EMs (EMs^-^) induced damages to yield-related parameters; however, soil application of EMs ameliorated salt stress-mediated damages to the yield. This was evidenced from the increased in number of fruits, average weight and cumulative yield of fruits by 14.6, 16.7, and 21.6%, respectively (**Table 2**). In comparison to hot pepper plants grown in salt-affected soil and supplemented with a low N dose (N_120_), the application of N at the rate of 150 or 180 kg ha^-1^ improved fruit number by 11.8 or 8.8%, fruit weight by 10.4 or 12.5%, and fruit yield by 12.6 or 15.5%, respectively (**Table 2**). However, the integration of EMs and N (particularly at higher levels) showed significant increases in the yield-related attributes. In this regards, the number, average weight and yield of fruits increased by 14.4 or 17.0%, 20.8 or 20.8%, and 28.4 or 27.5% in hot pepper plants treated with EMs^+^ × N_150_ or N_180_, respectively, compared to the EMs^-^ × N_150_ treatment (**Table 2**).

### 3.2 Plant water status, membrane integrity, and photosynthetic capacity

Growing season, EMs, N rates, and EMs × N level were induced modulation in hot pepper membrane integrity (MSI), water status (RWC), and photosynthetic capacity; SPAD chlorophyll, *Fv/Fm*, and PI (**Table 3**). The MSI, RWC, and SPAD chlorophyll were significantly influenced by the two growing seasons. However, there were no variations in the *Fv/Fm* or PI between the two growing seasons (**Table 3**).

For EMs application, hot pepper plants exposed to salinity stress and not treated with EMs markedly triggered a reduction in the aforementioned traits. Nevertheless, the application of EMs alleviated the salinity damage and elevated these parameters by 6.4, 5.6, 8.3, 8.0, and 10.0%, respectively (**Table 3**). For N fertilization, the results showed increasing N levels to 150 or 180 kg ha^-1^ in comparison to 120 kg ha^-1^, and significantly boosted the MSI by 4.4 or 4.7%, RWC by 5.6 or 10.5%, SPAD chlorophyll by 6.8 or 5.7%, *Fv/Fm* by 8.2 or 11.0%%, and PI by 19.1 or 19.1%%, orderly. Intriguingly, there were no appreciable differences between the N levels of 150 or 180 kg ha^-1^ (**Table 3**).

Regarding to the EMs × N interaction, the co-application of EMs and high levels of N fertilization improved the physio-biochemical attributes of salt-stressed hot pepper (**Table 3**). Compared to the EMs^–^ × N_150_ treatment, applying EMs along with N at 150 or 180 kg ha^-1^ increased the MSI (by 4.3 or 5.7%), RWC (by 10.5 or 11.5%), SPAD (by 4.9 or 5.4%), *Fv/Fm* (by 9.2 or 9.2%), and PI (by 11.3 or 11.3%), respectively (**Table 3**). Notably, in hot pepper plants treated with EMs^+^, there was no statistical differences between the two N levels of 150 or 180 kg ha^-1^ for the aforementioned physio-biochemical parameters (**Table 3**).

### 3.3 Total capsaicin, total protein, TSS, and proline contents

The proline, TSS, and total protein contents were higher in hot pepper plants grown during the spring season, while the total capsaicin content was similar in both growing seasons (**Table 4**). Salt-stressed hot pepper plants supplemented with EMs^+^ significantly increased the accumulation of total capsaicin by 11.6%, total protein by 12.5%, TSS by 7.4%, and proline by 20.0% more than the non-EMs-treated plants (**Table 4**). In saline soil, N supplied at a rate of 150 kg ha^-1^ significantly increased total capsaicin, total protein, TSS, and proline contents by 32.2, 8.3, 3.8, and 15.0%, respectively. While increasing N fertilization at 180 kg ha^-1^ further increased all previously mentioned attributes (except for proline) by 39.0, 12.5, 7.7, and 15.0%, respectively, as compared to those that received 120 kg ha^-1^ (**Table 4**).

The integrative effect of EMs and different N fertilization on the contents of total capsaicin, total protein, TSS, and proline were significant (**Table 4**). The integrative EMs^+^ × N_180_ resulted in higher values for the aforementioned attributes by 20.8, 16.7, 7.4, and 14.3%, respectively than the integrative EMs^-^ × N_150_. Whereas, the combined EMs^+^ × N_150_ treatment yielded the highest proline content followed by the combined EMs^+^ × N_180_ (**Table 4**).

### 3.4 GSH, AsA contents, and the activity of the enzymatic antioxidants

The contents of GSH and AsA in hot pepper plants were similar in the spring and autumn seasons (**Table 5**). EMs^+^ supplementation of salt-stressed hot pepper plants increased GSH by 29.4%, and AsA by 25.0% compared with the non-EMs-treated plants (**Table 5**). In comparison to low N fertilization (N_120_), higher N levels of N_150_ or N_180_ notably elevated the GSH by 33.3 or 53.3%, and AsA by 21.1 or 36.8% (**Table 5**).

Salt-stressed hot pepper plants supplemented with EMs^+^ and a high N rate of 180 kg ha^-1^ displayed higher GSH (by 44.4%), and AsA (by 38.1%) compared to non-supplemented plants with EMs and received recommended N dose (N_150_) (**Table 5**). The activities of APX, SOD, and GR were greater in hot pepper plants grown during the spring season, but the CAT activity was lower when compared to the enzymes activity in plants grown during the autumn season (**Table 5**). EMs-supplemented hot pepper plants showed greater activities of CAT, SOD, and GR than the salt-damaged hot pepper plants non- supplemented with EMs (**Table 5**). Regarding N fertilization, as N rates increased, the activities of all the analyzed antioxidant enzymes substantially increased (**Table 5**). Otherwise, the activities of CAT, SOD, and GR in the leaves of salt-stressed hot pepper were notably up-regulated by the ameliorating action of the integration EMs and N at N_150_ or N_180_, showing higher activities of these enzymes by 23.8 or 28.6%, 28.6 or 28.6%, and 66.7 or 77.8%, respectively, as compared to EMs^-^ × N_150_ treatment (**Table 5**).

### 3.5 Nutrients acquisition

Data of the nutrients acquisition in hot pepper plants in response to the growing season, EMs, N rate, and the EMs × N interaction are shown in **Table 6**. Hot pepper plants growing during the spring season showed higher P, K^+^, Ca^2+^, and Na^+^ contents, whereas both growing seasons showed similar N values. Application of EMs promoted the nutrients acquisition; N, P, K^+^, Ca^2+^, and Na^+^ in hot pepper plants than in non-applied EMs plants grown in saline soil. In contrast to hot pepper plants that received N_120_, fertilization at N_150_ or N_180_ significantly mediated increases in N by 10.6 or 18.0% and P by 11.1 or 13.9%, K^+^ by 6.7 or 6.6%, and Ca^2+^ by 9.2 or 13.1%, while decreased Na^+^ by 9.4 or 16.1%, respectively (**Table 6**).

The nutrients acquisition was significantly decreased in salt-stressed hot pepper untreated with EMs and fertilized with low N dosage (EMs^-^ × N_120_). However, co-application of EMs and higher N rates corrected these impairments in nutrient contents in hot pepper. The integrative EMs^+^ × N_150_ and EMs^+^ × N_180_ increased N (by 30.2 and 41.2%), P (by 13.2 and 5.3%), K^+^ (by 20.9 and 17.3%), Ca^2+^ (by 10.4 and 12.8%) associated with reduced Na^+^ (by 10.1 and 22.0%) contents, respectively, compared with the integrative EMs^-^ × N_150_ treatment (**Table 6**).

### 3.6 Leaf and stem anatomical structures

**Figure 1:**
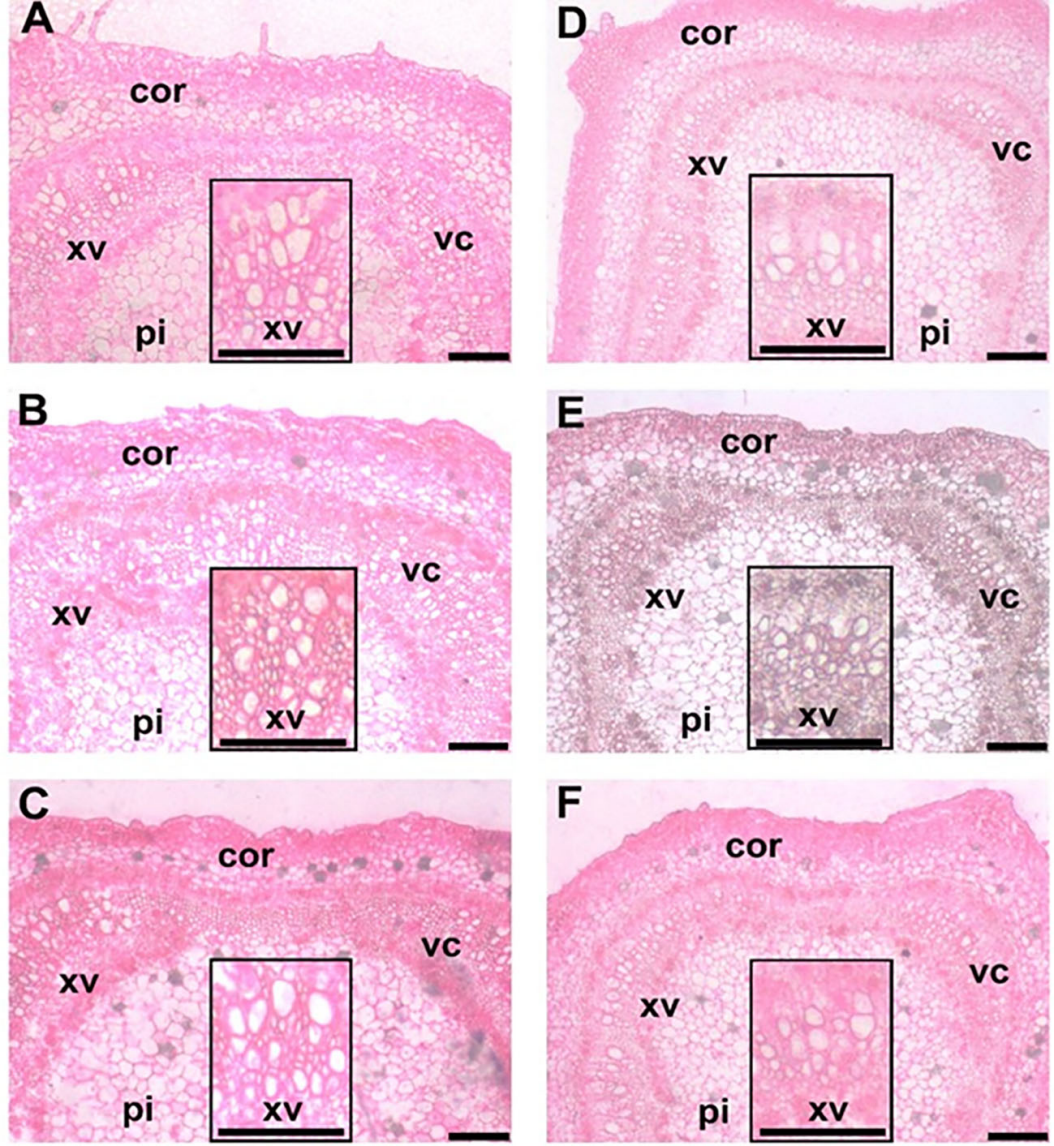
Stem transections of hot pepper (*Capsicum annum* L.) stems affected by the application of effective micoroorganisms and nitrogen under salt stress conditions. **(A)** EMs^+^ × N_120_; **(B)** EMs^+^ × N_150_; **(C)** EMs^+^ × N_180_; **(D)** EMs^-^ × N_120_; **(E)** EMs^-^ × N_150_; and **(F)** EMs^-^ × N_180_. EMs^-^/EMs+, without/with effective microorganisms application; N_120/150/180_,120/150/180 kg nitrogen ha^-1^; Cor, cortex; xv, xylem vessels; pi: pith; vc: vascular cylinder. Bars = 200 µm.

In gereal, the data indicated that saline soil caused a negative influence on the anatomical features of the stems and leaves of hot pepper plants. However, the application of EMs and/or higher N dosages (N_150_ and N_180_) reversed the previous pattern and mediated improvements in the anatomical features in hot pepper (**Figure 1**).

**Figure 2:**
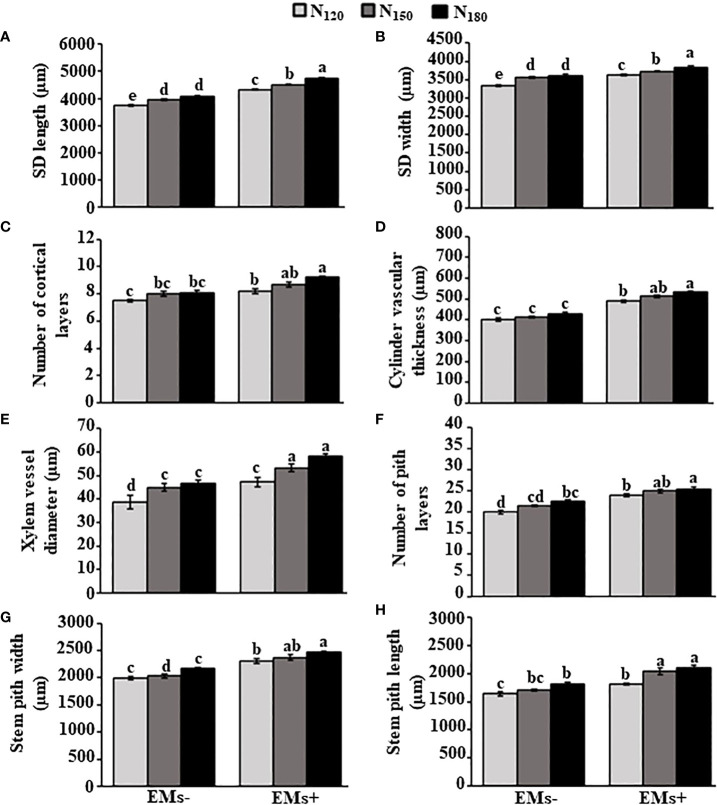
Integrative effect of effective microorganisms and nitrogen on the stem anatomical structures of hot pepper (*Capsicum annum* L.) plants grown in saline soil. **(A)** stem dimension (SD) length; **(B)** stem dimension (SD) width; **(C)** number of cortical layers; **(D)** cylinder vascular thickness; **(E)** xylem vessel diameter; **(F)** number of pith layers; **(G)** stem pith width; and **(H)** stem pith length of pepper plants grown in saline soil. The vertical bar represents the standard error. Different letters on the bar indicate a significant difference by LSD at *P* ≤ 0.05. EMs^-^/EMs+, without/with effective microorganisms application; N_120/150/180_,120/150/180 kg nitrogen ha^-1^; SD, stem dimension.

In comparison to the combined EMs^-^ with recommended N dose (N_150_), the combined EMs^+^ **×** N_150_ or EMs^+^ **×** N_180_ showed substantial improvements in the stem dimensions, including length by 14.0 or 19.7% (**Figure 2A**) and width by 4.5 or 7.9% (**Figure 2B**), respectively. In addition, there was an increase by 8.7 or 15.0% in the number of cortical layers (**Figure 2C**), and 24.2 or 29.1% in the thickness of the vascular cylinder (**Figure 2D**) when hot pepper plants were treated with EMs^+^ **×** N_150_ or EMs^+^ **×** N_180_, respectively, compared with that in EMs^-^ **×** N_150_. The same treatments of EMs^+^ with either high levels of N (N_150_ or N_180_) also increased xylem vessel diameter, number of pith layers, pith width and length up to 18.4 or 29.6% (**Figure 2E**), 16.3 or 18.6% (**Figure 2F**), 16.7 or 21.3% (**Figure 2G**), and 19.5 or 23.4% (**Figure 2H**), respectively, in comparison of EMs^-^ combined with N_150_.

**Figure 3:**
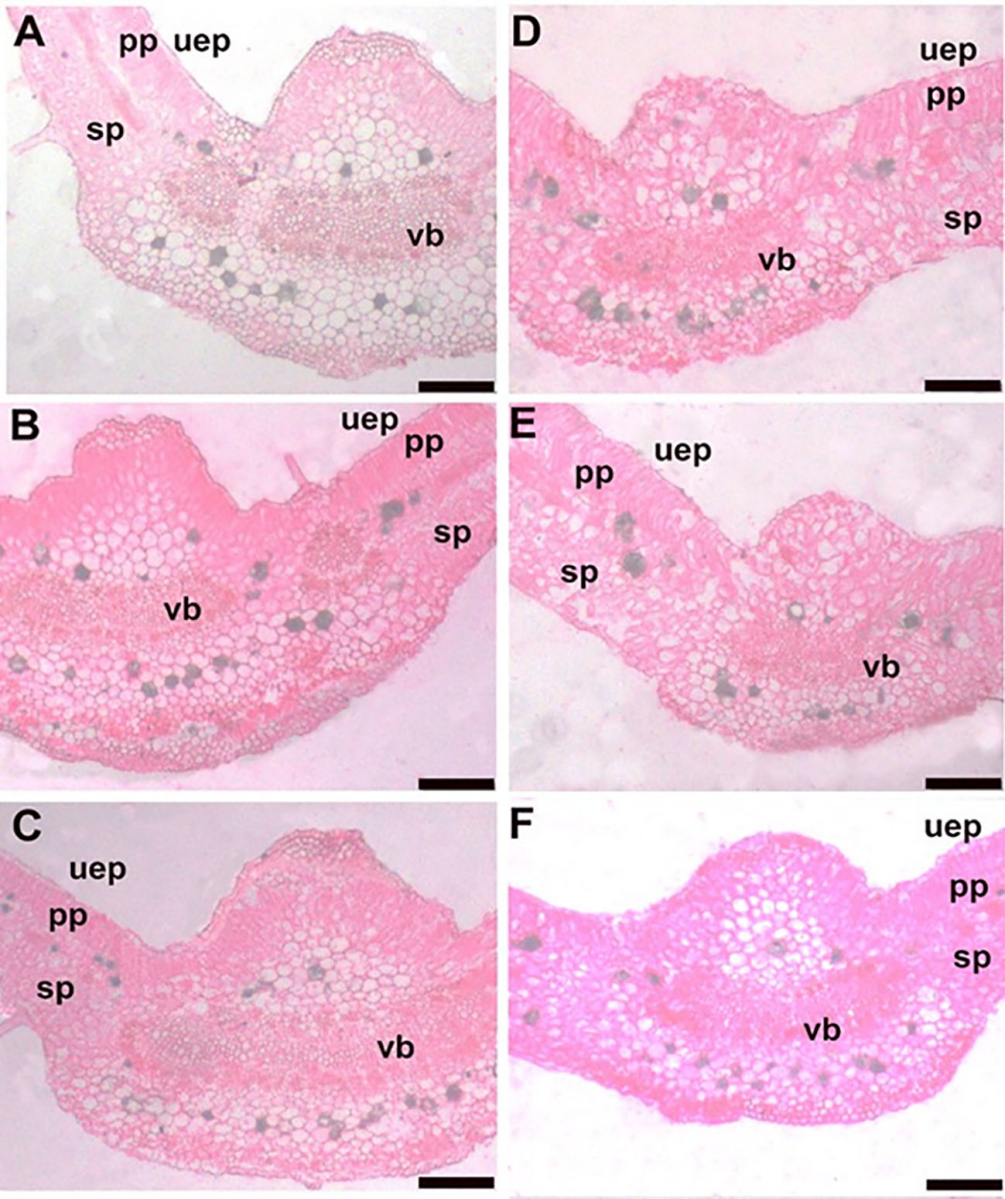
Transections in the midvein of leaves of hot pepper (*Capsicum annum* L.) plants affected by the application of effective microorganisms and nitrogen under salt stress conditions. **(A)** EMs^+^ × N_120_; **(B)** EMs^+^ × N_150_; **(C)** EMs^+^ × N_180_; **(D)** EMs^-^ × N_120_; **(E)** EMs^-^ × N_150_; and **(F)** EMs^-^ × N_180_. Bars = 200 µm. EMs^-^/EMs+, without/with effective microorganisms application; N_120/150/180_,120/150/180 kg nitrogen ha^-1^; uep: upper epidermis; sp: sponge parenchyma; pp: plastid parenchyma; vb: vascular bundle of the midvein.

**Figure 4:**
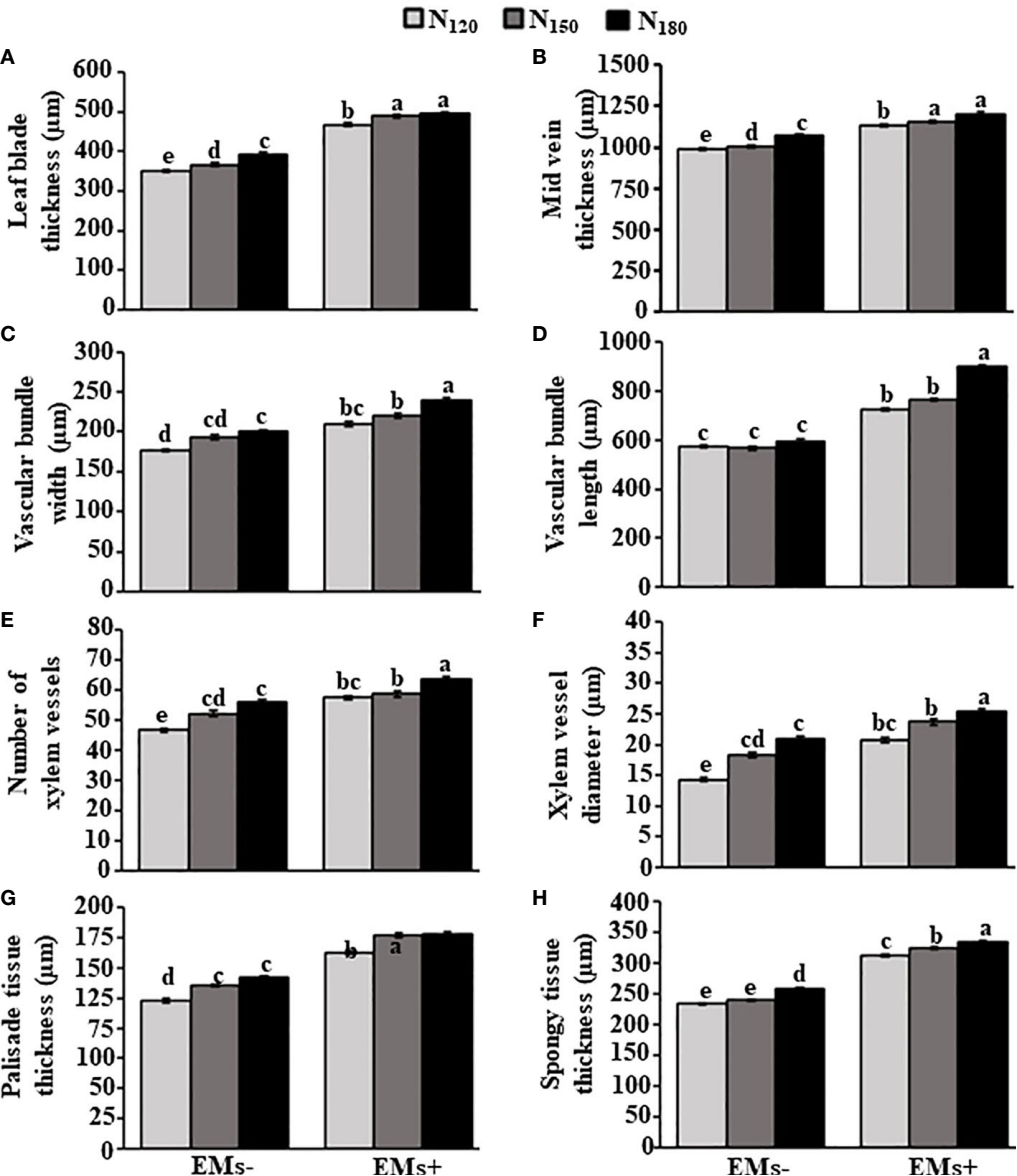
Integrative effect of effective microorganisms and nitrogen on the leaf anatomical structures of hot pepper (*Capsicum annum* L.) plants grown in saline soil. **(A)** leaf blade thickness, **(B)** mid vein thickness, **(C)** vascular bundle width, **(D)** vascular bundle length, **(E)** number of xylem vessels, **(F)** diameter of xylem vessels, **(G)** thickness of palisade tissue, and **(H)** thickness of spongy tissue of hot pepper plants grown in saline soil. The vertical bar represents the standard error. Different letters on the bar indicate a significant difference by LSD at *P* ≤ 0.05. EMs^-^/EMs+, without/with effective microorganisms application; N_120/150/180_,120/150/180 kg nitrogen ha^-1^.

Similarly, the anatomy of leaf features was altered in response to EMs^+^ and N_150_ or N_180_ fertilization (**Figure 3**). In this regard, application of EMs^+^ **×** N_150_ or EMs^+^ **×** N_180_ increased the leaf blade thickness and mid vein thickness by 33.6 or 35.5% (**Figure 4A**) and 14.9 or 19.9% (**Figure 4B**), respectively, when compared to EMs^-^ × N_150_ treatment. In addition, the width of vascular bundles increased by 13.8 or 24.2% (**Figure 4C**), the length of the leaf vascular bundles increased by 35.3 or 58.8% (**Figure 4D**) in plants treated with the combination of EMs^+^ and N_150_ or EMs^+^ and N_180_, respectively, compared to EMs^-^ × N_150_ treatment. In comparison with EMs^-^ × N_150_, the number and diameter of leaf xylem vessels also improved by 12.7 or 21.9% (**Figure 4E**), and 29.5 or 38.3% (**Figure 4F**), in N_150_ or N_180_ combined with EMs^+^, respectively. We also observed an increase in the thickness of palisade and spongy tissues in EMs^+^ **×** N_150_ or EMs^+^ **×** N_180_ by 31.0 or 31.7% (**Figure 4G**), and 35.26 or 39.0% (**Figure 4H**), respectively when compared to EMs^-^ × N_150_ treatment.

## 4 Discussion

Soil salinity is recognized as one of the main abiotic conditions that cause extensive losses to agricultural production worldwide (Numan et al., 2018). Soil salinization is a serious threat to vegetable production, especially in arid regions like Egypt, where the majority of newly reclaimed soils are salt-affected (Osman and Rady, 2012). Salinity stress can lower the osmotic potential of the soil solution causing physiological drought, oxidative damage, and nutritional deficiency in plants. Together, these factors inhibit many physio-biochemical, anatomical, and metabolic processes such as photosynthetic pigments, gas exchange, transpiration, protein expression, and hormone metabolism (Arif et al., 2020; Rady et al., 2021b). This may also result in the loss of membrane integrity, cell turgor, disturbance in plant-water relations, and reduced meristic activity, cell elongation and expansion; thus, mediated impairment in plant growth traits and yield shown in the current study can be considered as an evidence (**Table 2**).

Plants, on the other hand, possess multiple mechanisms to counteract the inhibitory effect of salinity through ion homeostasis and compartmentalization, transport, ion uptake, osmotic adjustments, and stimulation of antioxidative defense system (Gupta and Huang, 2014; Semida et al., 2021a). Under severe stress conditions, plant tolerance cannot relieve salinity stress as a result of an imbalance between the production of antioxidants, and small-molecular-weight substances, and the suppression of oxidative stress biomarkers (Rady et al., 2021a). Therefore, biostimulants, such as EMs and N enrichment, may increase tolerance to salinity stress in crop plants.

The integration of EMs with N fertilization has not been applied previously; and research focusing on the modulation in the physio-biochemical, agronomic and anatomical changes associated with salinity stress has not been studied yet. In the current study, soils treated with EMs and N increased the accumulation of osmoprotectant substances (proline and TSS) and improved the antioxidative defense system of salt-stressed hot pepper. These actions mediated the positive osmoregulatory modulation, counteracted oxidative stress, simultaneously stimulated nutrient acquisition, recovered biosynthesis of photosynthetic pigments, and increased PSII activity; thus, resulting in improved hot pepper performance.

The application of EMs and/or 150 or 180 kg N unit ha^-1^ notably enhanced hot pepper performance planted under saline soil conditions (EC 9.6 dS m^−1^; **Table 1**). These strategies stimulated hot pepper growth (*e.g.* plant height, leaf number, number of branches, leaf area, and shoot dry weight), and fruit yield (**Table 2**). Similarly, EMs-amended saline soil improved the sweet potato and tomato growth parameters, and productivity under high salinity levels (Ndona et al., 2011; Abd El-Mageed et al., 2022). Such response can be attributed to the synthesis of bioactive substances, such as amino acids, sugars, hormones, lactic acid, vitamins, and enzymes (Hu and Qi, 2013; Talaat et al., 2015). The enhancement in oat growth traits and grain yield in response to N supplementation has also been reported (Song et al., 2019). The growth and development of a plant are primarily dependent on N, as a critical component of metabolically active compounds such as chlorophyll, protein, nucleic acids, and enzymes (Poudel et al., 2018). This recovery in growth, biomass production, and fruit yield of salt-stressed hot pepper achieved by supplementing plants with EMs and/or high N rates may regulate mechanisms in relieving the salt-induced stress.

**Table 1:** Analysis of physicochemical proprieties of the experimental soil.

Property	Value
Physical	
Particle size distribution:	
Sand	79.20
Silt (%)	10. 00
Clay (%)	10.80
Texture class	Sandy loam
Bulk density (g cm^-3^)	1.59
Hydraulic conductivity (K_sat_) (cm h^-1^)	2.22
Field capacity	19.33
Wilting point (%)	9.73
Available water (%)	9.60
Chemical	
pH [soil:water (w/v) ratio of 1:2.5]	7.76
ECe [at a soil - paste extract] (dS m^-1^)	9.6
CEC (cmol kg^-1^)	11.00
CaCO_3_ (%)	8.6
OM	0.98
ESP (%)	10.62
SAR	12.07
Available nutrients	
N (%)	0.04
P (%)	4.38
K (%)	46.9
Fe (mg kg^-1^)	2.42
Mn (mg kg^-1^)	6.61
Zn (mg kg^-1^)	0.81
Cu (mg kg^-1^)	0.62

ECe, electrical conductivity of soil saturated paste extract; CEC, cation exchange capacity; OM, organic matter; ESP, exchangeable sodium percentage; SAR, sodium adsorption ratio.

**Table 2:** Effect of effective microorganisms and nitrogen levels on growth characteristics and yield related-parameters of hot pepper (*Capsicum annum* L.) plants grown in saline soil in spring and autumn seasons.

Treatments		Plant height (cm)	Number of leaves plant^-1^	Number of branches plant^-1^	Leaf area (dm^2^ plant^-1^)	Shoot DW (g plant^-1^)	Fruit number plant^-1^	Fruit weight (g)	Fruit yield (t ha^-1^)
**Seasons**		*	**	NS	**	**	NS	NS	**
SS		61.5 ± 1.4a	94.3 ± 5.1a	7.6 ± 0.35a	32.7 ± 2.0a	43.2 ± 1.3a	117.3 ± 2.8a	5.1 ± 0.12a	12.6 ± 0.44a
AS		58.1 ± 1.1b	36.6 ± 1.1b	7.8 ± 0.37a	28.2 ± 1.8b	37.4 ± 1.6b	116.8 ± 2.6a	5.2 ± 0.14a	10.0 ± 0.27b
**EMs**		**	**	*	**	**	**	**	**
EMs^-^		57.1 ± 1.3b	56.1 ± 5.8b	7.3 ± 0.31b	27.1 ± 1.8b	36.3 ± 1.3b	109.1 ± 1.9b	4.8 ± 0.10b	10.2 ± 0.32b
EMs^+^		62.5 ± 1.0a	74.8 ± 9.0a	8.1 ± 0.38a	33.8 ± 1.8a	44.4 ± 1.3a	125.0 ± 1.8a	5.6 ± 0.10a	12.4 ± 0.46a
**N levels**		**	**	**	**	**	*	**	**
N_120_		54.8 ± 1.1b	53.9 ± 7.0b	6.4 ± 0.38b	24.5 ± 0.92c	35.2 ± 1.4b	109.6 ± 2.9b	4.8 ± 0.11b	10.3 ± 0.38b
N_150_		61.4 ± 1.1a	72.1 ± 10.8a	8.1 ± 0.38a	39.2 ± 1.7a	43.5 ± 2.0a	122.5 ± 3.0a	5.3 ± 0.17a	11.6 ± 0.63a
N_180_		63.2 ± 1.5a	70.3 ± 10.2a	8.6 ± 0.29a	27.6 ± 1.8b	42.4 ± 1.5a	119.2 ± 2.7a	5.4 ± 0.15a	11.9 ± 0.62a
**EMs × N**		**	**	*	**	**	*	**	**
EMs^-^	N_120_	52.5 ± 1.6c	46.3 ± 8.0d	5.7 ± 0.49c	23.6 ± 1.2c	32.9 ± 1.3c	101.4 ± 2.3c	4.5 ± 0.12c	9.5 ± 0.32d
	N_150_	58.3 ± 0.5b	60.8 ± 11.3c	8.0 ± 0.45ab	35.9 ± 2.6b	38.8 ± 2.7b	111.2 ± 2.9b	4.8 ± 0.13bc	10.2 ± 0.54c
	N_180_	60.5 ± 0.9b	61.0 ± 11.4c	8.2 ± 048ab	21.7 ± 0.86c	38.0 ± 1.5b	114.8 ± 2.3b	5.0 ± 0.11b	10.8 ± 0.69bc
EMs^+^	N_120_	57.2 ± 1.2b	61.5 ± 11.4c	7.2 ± 0.40b	25.4 ± 1.4c	38.2 ± 1.7b	117.8 ± 2.4b	5.1 ± 0.10b	11.1 ± 0.51b
	N_150_	64.5 ± 2.3a	86.3 ± 18.4a	8.2 ± 0.65ab	42.4 ± 1.3a	48.2 ± 1.4a	127.2 ± 2.6a	5.8 ± 0.14a	13.1 ± 0.79a
	N_180_	65.8 ± 1.3a	79.5 ± 17.3b	9.0 ± 0.26a	43.5 ± 0.65a	46.8 ± 0.6a	130.1 ± 1.8a	5.8 ± 0.10a	13.0 ± 0.84a

Data represent the single effects of growing seasons, EMs, N levels, or the interaction effect of EMs **×** N levels. Average values in each column followed by the same letters indicate no significant difference at P ≤ 0.05 using Fisher’s least-significant difference test. * and ** represent significant differences at P ≤ 0.05 and P ≤ 0.01, respectively. NS, no significant difference at P = 0.05.

DW, dry weight; SS, spring season; AS, autumn season; EMs^-^/EMs+, without/with effective microoragisms application; N_120/150/180_,120/150/180 kg nitrogen ha^-1^.

The RWC is an important indicator of leaf water status that reflects the physiological activities in the plant; whereas the MSI is an indicator of the degree of membrane integrity (Petrov et al., 2018; Abdelkhalik et al., 2019; Semida et al., 2019). Nevertheless, both RWC and MSI were decreased in response to salt stress in the current study (**Table 3**). However, when EMs^+^ **×** N_150_ or EMs^+^ **×** N_180_ was applied to the soil cultivated with hot pepper plants, amelioration of the salt-induced damage in tissue water content and membrane integrity was observed. Recovery of RWC in plant cells by the application of EMs^+^ **×** N_150_ or EMs^+^ **×** N_180_ could be due to the effect of EMs^+^ and/or high N levels in increasing the accumulation of osmoprotectants (proline and TSS; **Table 4**), and K^+^ ion (**Table 6**) as an osmotic adjustment mechanism for salt stress tolerance (Desoky et al., 2021). These could result in the continuity of metabolic activities, stomatal conductance, gas exchange, CO_2_ assimilation, and photosynthesis in plant tissues by osmoregulation and other adaptive processes (Lawlor and Cornic, 2002; Slabbert and Krüger, 2014). Moreover, our findings revealed restoration in integrity and stability of the leaf tissue cell in response to EMs^+^ **×** N_150_ or EMs^+^ **×** N_180_, associated with the up-regulation of antioxidative compounds (**Table 5**), which also helps mitigate the oxidative damage and preserve the membrane in a healthy state (Ahanger et al., 2019; Abd El-Mageed et al., 2022).

**Table 3:** Effect of effective microorganisms and nitrogen levels on membrane stability index, relative chlorophyll content, soil-plant-analysis development value, and chlorophyll fluorescence (*F_v_/F_m_* and PI) of hot pepper (*Capsicum annum* L.) plants grown in saline soil in spring and autumn seasons.

Treatments		MSI (%)	RWC (%)	SPAD value	*F_v_/F_m_*	PI
**Seasons**		**	**	**	NS	NS
SS		79.3 ± 1.1a	74.2 ± 1.8a	69.6 ± 1.4a	0.78 ± 0.01a	5.3 ± 0.14a
AS		65.6 ± 07b	69.8 ± 1.3b	66.0 ± 1.5b	0.77 ± 0.01a	5.3 ± 0.16a
**EMs**		**	**	**	**	**
EMs^-^		70.2 ± 1.6b	70.0 ± 1.8b	65.1 ± 0.9b	0.75 ± 0.01b	5.0 ± 0.13b
EMs^+^		74.7 ± 2.0a	73.9 ± 1.0a	70.5 ± 1.5a	0.81 ± 0.01a	5.5 ± 0.15a
**N** levels		**	**	*	**	**
N_120_		70.3 ± 2.1b	68.3 ± 1.2b	65.1 ± 1.8b	0.73 ± 0.01b	4.68 ± 0.12b
N_150_		73.4 ± 2.3a	72.1 ± 1.9ab	69.5 ± 1.2a	0.79 ± 0.01a	5.6 ± 0.14a
N_180_		73.6 ± 2.5a	75.5 ± 1.9a	68.8 ± 0.7a	0.81 ± 0.01a	5.6 ± 0.15a
**EMs × N**		**	**	**	**	**
EMs^-^	N_120_	67.89 ± 2.8d	66.94 ± 1.8d	61.53 ± 0.67c	0.71 ± 0.01c	4.5 ± 0.15c
	N_150_	72.05 ± 3.2bc	68.53 ± 3.0cd	67.87 ± 0.70ab	0.76 ± 0.01b	5.3 ± 0.15ab
	N_180_	70.60 ± 2.6cd	74.62 ± 3.8abc	66.00 ± 1.01b	0.78 ± 0.01b	5.36 ± 0.18ab
EMs^+^	N_120_	72.62 ± 3.2bc	69.66 ± 1.6bcd	68.65 ± 2.9ab	0.75 ± 0.01b	4.8 ± 0.16bc
	N_150_	75.17 ± 3.4ab	75.71 ± 1.3ab	71.20 ± 2.3a	0.83 ± 0.01a	5.9 ± 0.14a
	N_180_	76.18 ± 4.3a	76.40 ± 0.9a	71.55 ± 1.4a	0.83 ± 0.01a	5.9 ± 0.22a

Data represent the single effects of growing seasons, EMs, N levels, or the interaction effect of EMs **×** N levels. Average values in each column followed by the same letters indicate no significant difference at P ≤ 0.05 using Fisher’s least-significant difference test. * and ** represent significant differences at P ≤ 0.05 and P ≤ 0.01, respectively. NS, no significant difference at P = 0.05. MSI, membrane stability index; RWC, relative chlorophyll content; SPAD, soil-plant-analysis development; F_v_/F_m_, maximum quantum yield of photosystem II; PI, performace index; SS, spring season; AS, autumn season; EMs^-^/EMs+, without/with effective microoragisms application; N_120/150/180_,120/150/180 kg nitrogen ha^-1^.

**Table 4:** Effect of effective microorganisms and nitrogen levels on total capsaicin, total protein, total soluble sugars, and proline contents of hot pepper (*Capsicum annum* L.) plants grown in saline soil in spring and autumn seasons.

Treatments		Total capsaicin	Total protein	Total soluble sugars	Proline
		(mg g^-1^ DW)		(mg g^-1^ FW)	
**Seasons**		NS	*	*	*
SS		0.72 ± 0.03a	2.6 ± 0.38a	2.9 ± 0.14a	2.3 ± 0.01a
AS		0.73 ± 0.03a	2.4 ± 0.38b	2.7 ± 0.15b	2.1 ± 0.01b
**EMs**		**	**	**	**
EMs^-^		0.69 ± 0.02b	2.4 ± 0.35b	2.7 ± 0.12b	2.0 ± 0.01b
EMs^+^		0.77 ± 0.03a	2.7 ± 0.31a	2.9 ± 0.12a	2.4 ± 0.01a
**N levels**		**	**	**	**
N_120_		0.59 ± 0.01c	2.4 ± 0.20c	2.6 ± 0.10c	2.0 ± 0.01b
N_150_		0.78 ± 0.02b	2.6 ± 0.28b	2.7 ± 0.11b	2.3 ± 0.00a
N_180_		0.82 ± 0.02a	2.7 ± 0.26a	2.8 ± 0.11a	2.3 ± 0.001a
**EMs × N**		**	**	**	**
EMs^-^	N_120_	0.58 ± 0.02e	2.3 ± 0.09e	2.6 ± 0.03e	1.8 ± 0.01e
	N_150_	0.72 ± 0.00d	2.4 ± 0.10d	2.7 ± 0.02d	2.1 ± 0.01d
	N_180_	0.7 ± 0.01c	2.5 ± 0.12c	2.7 ± 0.02d	2.2 ± 0.01c
EMs^+^	N_120_	0.59 ± 0.01e	2.5 ± 0.11c	2.8 ± 0.04c	2.2 ± 0.01c
	N_150_	0.84 ± 0.01b	2.7 ± 0.10b	2.8 ± 0.06b	2.5 ± 0.00a
	N_180_	0.87 ± 0.01a	2.8 ± 0.12a	2.9 ± 0.05a	2.4 ± 0.01b

Data represent the single effects of growing seasons, EMs, N levels, or the interaction effect of EMs **×** N levels. Average values in each column followed by the same letters indicate no significant difference at P ≤ 0.05 using Fisher’s least-significant difference test. * and ** represent significant differences at P ≤ 0.05 and P ≤ 0.01, respectively. NS, no significant difference at P = 0.05.DW/FW, dry/fresh weight; SS, spring season; AS, autumn season; EMs^-^/EMs+, without/with effective microoragisms application; N_120/150/180_,120/150/180 kg nitrogen ha^-1^.

**Table 5:** Effect of effective microorganisms and nitrogen levels on non-enzymatic and enzymatic antioxidants of hot pepper (*Capsicum annum* L.) plants grown in saline soil in spring and autumn seasons.

		Non-enzymatic content		Enzymatic activities			
Treatments		Glutathione	Ascorbic acid	CAT	APX	SOD	GR
		(mg g^-1^ FW)		(µmol mg^-1^ protein)			
**Seasons**		NS	NS	*	**	*	**
SS		0.20 ± 0.01a	0.22 ± 0.01a	0.22 ± 0.01b	0.18 ± 0.01a	0.22 ± 0.01a	0.14 ± 0.01a
AS		0.19 ± 0.01a	0.23 ± 0.01a	0.23 ± 0.01a	0.16 ± 0.1b	0.21 ± 0.01b	0.11 ± 0.01b
EMs		**	**	**	NS	**	**
EMs^-^		0.17 ± 0.01b	0.20 ± 0.01b	0.20 ± 0.01b	0.17 ± 0.01a	0.19 ± 0.00b	0.10 ± 0.01b
EMs^+^		0.22 ± 0.01a	0.25 ± 0.01a	0.25 ± 0.01a	0.17 ± 0.01a	024 ± 0.01a	0.15 ± 0.00a
**N levels**		**	**	**	**	**	**
N_120_		0.15 ± 0.01c	0.19 ± 0.01c	0.19 ± 0.01c	0.16 ± 0.01c	0.21 ± 0.01b	0.11 ± 0.01c
N_150_		0.20 ± 0.01b	0.23 ± 0.01b	0.23 ± 0.01b	0.17 ± 0.01b	0.22 ± 0.01a	0.12 ± 0.01b
N_180_		0.23 ± 0.01a	0.26 ± 0.01a	0.25 ± 0.01a	0.19 ± 0.01a	0.23 ± 0.01a	0.14 ± 0.01a
**EMs × N**		**	**	**	**	**	**
EMs^-^	N_120_	0.14 ± 0.01e	0.17 ± 0.00e	0.17 ± 0.00c	0.16 ± 0.02c	0.19 ± 0.00d	0.08 ± 0.01e
	N_150_	0.18 ± 0.00d	0.21 ± 0.00d	0.21 ± 0.02b	0.14 ± 0.01e	0.19 ± 0.01d	0.09 ± 0.01d
	N_180_	0.20 ± 0.00c	0.22 ± 0.00c	0.22 ± 0.00b	0.20 ± 0.01a	0.21 ± 0.01c	0.11 ± 0.01c
EMs^+^	N_120_	0.16 ± 0.01de	0.20 ± 0.01d	0.22 ± 0.00b	0.15 ± 0.02d	022 ± 0.02b	0.14 ± 0.00b
	N_150_	0.23 ± 0.00b	0.26 ± 0.00b	0.26 ± 0.01a	0.18 ± 0.02b	0.25 ± 0.00a	0.15 ± 0.01b
	N_180_	0.26 ± 0.01a	0.29 ± 0.01a	0.27 ± 0.01a	0.18 ± 0.01b	0.25 ± 0.01a	0.16 ± 0.01a

Data represent the single effects of growing seasons, EMs, N levels, or the interaction effect of EMs **×** N levels. Average values in each column followed by the same letters indicate no significant difference at P ≤ 0.05 using Fisher’s least-significant difference test. * and ** represent significant differences at P ≤ 0.05 and P ≤ 0.01, respectively. NS, no significant difference at P = 0.05. CAT, catalase; APX, ascorbate peroxidase; SOD, superoxide dismutase; GR, glutathione reductase; FW, fresh weight; SS, spring season; AS, autumn season; EMs^-^/EMs+, without/with effective microoragisms application; N_120/150/180_,120/150/180 kg nitrogen ha^-1^.

**Table 6:** Effect of effective microorganisms and nitrogen levels on the nutrient contents in the leaves of hot pepper (*Capsicum annum* L.) plants grown in saline soil in spring and autumn seasons.

Treatments		N	P	K^+^	Ca^2+^	Na^+^
		(mg g^-1^ DW)				
**Seasons**		NS	*	*	*	*
SS		20.7±1.16a	4.0±0.38a	14.69±0.34a	16.10±0.27a	12.73±0.38a
AS		20.6±0.63a	3.8±0.38b	13.60±0.47b	15.07±0.35b	12.07±0.42b
**EMs**		**	**	**	**	**
EMs^-^		17.7±0.54a	3.8±0.31b	13.02±0.34b	14.82±0.32b	13.44±0.34a
EMs^+^		23.3±0.60b	4.0±0.35a	15.28±0.32a	16.36±0.23a	11.36±0.30b
**N levels**		**	**	*	**	**
N_120_		18.9±1.06c	3.6±0.20c	13.55±0.54b	14.51±0.39c	13.55±0.43a
N_150_		20.9±1.01b	4.0±0.28b	14.46±0.54a	15.84±0.33b	12.28±0.35b
N_180_		22.3±1.17a	4.1±0.26a	14.44±0.48a	16.41±0.30a	11.37±0.50c
**EMs × N**		**	**	**	**	**
EMs^-^	N_120_	15.9±0.57e	3.6±0.09c	12.43±0.73d	13.56±0.53d	14.76±0.26a
	N_150_	18.2±0.79d	3.8±0.10c	13.09±0.66cd	15.06±0.42c	12.93±0.46b
	N_180_	18.9±1.02d	4.1±0.12ab	13.53±0.25c	15.83±0.25b	12.64±0.60b
EMs^+^	N_120_	21.9±1.01c	3.6±0.11c	14.67±0.48b	15.47±0.18bc	12.35±0.38bc
	N_150_	23.7±0.95b	4.3±0.10a	15.82±0.29a	16.62±0.25a	11.63±0.41c
	N_180_	25.7±0.58a	4.0±0.12b	15.36±0.78ab	16.99±0.46a	10.09±0.29d

Data represent the single effects of growing seasons, EMs, N levels, or the interaction effect of EMs **×** N levels. Average values in each column followed by the same letters indicate no significant difference at P ≤ 0.05 using Fisher’s least-significant difference test. * and ** represent significant differences at P ≤ 0.05 and P ≤ 0.01, respectively. NS, no significant difference at P = 0.05. N, nitrogen; P, phossphorus; K, potassium; Ca, calcium; Na, sodium; DW, dry weight; SS, spring season; AS, autumn season; EMs^-^/EMs+, without/with effective microoragisms application; N_120/150/180_,120/150/180 kg nitrogen ha^-^

Salinity stress exacerbates free radical generation, nutrient deficiency, and osmotic stress; thus, leading to the degradation of chlorophyll *via* the chlorophyllase enzyme, reduction in chlorophyll biosynthesis, induction of photoinhibition to the PSII (*Fv/Fm*, and PI), and decreased SPAD chlorophyll values, as evidenced in the current study (Guidi et al., 2019; Akhter et al., 2021). Our results also indicated that the application of EMs^+^ and/or high N levels enhanced SPAD chlorophyll values, and the efficiency of PSII (*Fv/Fm*, and PI). This suggests that the amount of light-harvest by hot pepper plants for photosynthesis can be increased, as suggested by Zhao et al. (2017). Furthermore, increasing the photosynthetic machinery and relative chlorophyll content under salt stress after the application of EMs^+^ and/or high N gives biochemical insights into salt stress tolerance (Ashraf and Harris, 2013; Ahmed et al., 2022). Similar results have been obtained when EMs (Abd El-Mageed et al., 2022) and N (Ahanger et al., 2019) elevated the compatible solutes accumulation, antioxidant capacity, and nutrients acquisition in sweet potato and wheat, to prevent oxidative damage, preserve cell water status and membrane stability, protecting the photosynthetic transport electron, increasing chlorophyll contents, and the efficiency of photosynthesis. N supplementation also enhanced the photosynthetic capacity *via* the activity of Rubisco, causing significant alleviation of salinity-induced reduction of photosynthesis (Iqbal et al., 2015).

In the present study, hot pepper plants treated with EMs^+^ **×** N_150_ or EMs^+^ **×** N_180_ exhibited higher capacity to accumulate osmoregulators of proline and TSS for cellular osmotic hemostasis (**Table 4**). The production of osmolytes as a protective mechanism under salinity stress is mostly linked to EMs and N, and salt stress tolerance (Iqbal et al., 2015; Talaat et al., 2015). These osmolytes-mediated osmotic balance, stimulate water uptake, increase RWC, stabilize cellular membranes and proteins against the damaging effect of free radicals (Semida et al., 2020; Wang et al., 2022). These findings demonstrated the ameliorative effect of EMs and high N levels on hot pepper plants against salt stress. Our data clearly illustrated that the application of EMs^+^ **×** N_150_ or EMs^+^ **×** N_180_ further increased soluble protein (**Table 4**). The N-supply to wheat (*Triticum aestivum*) elevated the activity of nitrate reductase, allowing it to be rapidly converted into N precursors for amino acids biosynthesis and proteins (Ahanger et al., 2019). The data in the current study were consistent with those of Talaat (2015) who reported that EMs activated the nitrate uptake and induced the nitrate reductase activity, resulting in increased protein content in common bean (*Phaseolus vulgaris*) grown in saline conditions. Capsaicin is an alkaloid compound responsible for the pungency/spiciness flavor of hot peppers (Barbero et al., 2014), which was also increased in hot pepper plants supplemented with EMs and/or high N levels (**Table 4**).

Under salinity stress conditions, plants develop defense mechanisms, including the synthesis of enzymes and non-enzymes antioxidants to protect their cells from oxidants. Large pools of these antioxidative compounds are potent ROS scavengers that govern redox homeostasis and restore cellular functions under salt stress (Kapoor et al., 2015; Kaya et al., 2020b). The present study showed that applying EMs and/or high N levels substantially increased the activity of CAT, SOD, and GR along with the increased accumulation of AsA, and GSH in hot pepper plants grown under salinity stress (**Table 5**). This indicates that EMs and/or high N levels may ameliorate the oxidative damage through enhancing the antioxidant enzymes and improving the ascorbate-glutathione cycle. Similarly, EMs-treated sweet potato (*Ipomoea batatas*) cultivated in saline soil exhibited higher accumulations of AsA, GSH, CAT, SOD, GR, and POD (Abd El-Mageed et al., 2022). Furthermore, N-supply to the growing media of NaCl-stressed cotton seedlings mediated increases in the antioxidants (CAT, SOD, and POD) by 3.5 folds to boost salt tolerance (Anjum et al., 2012).

Our data revealed that soil salinity caused a negative influence on hot pepper plants as indicated by increased levels of Na^+^, and decreased levels of N, P, K^+^, and Ca^2+^ in leaves (**Table 6**); thus, this might trigger the ionic imbalance, and nutrient deficiency (Sitohy et al., 2020). Nonetheless, EMs^+^ **×** N_150_ or EMs^+^ **×** N_180_ could “correct” these perturbations in ion homeostasis by increasing N, P, K^+^, and Ca^2+^ but decreasing Na^+^ acquisition as another mechanism of salt tolerance by which hot pepper plants use to maintain ion balance and cell turgor (Wang et al., 2022). These favorable impacts on nutrients accumulation in hot pepper could be related to the role of EMs and/or N in increasing cell membrane integrity; thus, increasing selectivity and transport of ions, besides the accumulation of osmotic regulatory compounds (**Table 4**), and K^+^ (**Table 6**) for increasing water and nutrients influx (Talaat et al., 2015; Sikder et al., 2020).

Abiotic stress affects molecular, physiological, and biochemical processes in plants, resulting in the alternations in morphological structures and disturbance in plant performance (Mangena, 2018; Abd El-Mageed et al., 2021; Semida et al., 2021b). Exposure to salinity stress caused negative changes in leaf and stem anatomical structures (**Figure 2**; **Figure 4**). However, the application of EMs and/or N mediated the recovery in such features. Similarly, EMs improved anatomical structures of hot pepper plants under salt stress (Abd El-Mageed et al., 2020). Overall, we argue that our findings indicate a useful link between the application of EMs and/or N and salt stress tolerance in hot pepper plants.

## 5 Conclusion

The obtained results from this study revealed that the application of EMs^+^ **×** N_150_ or EMs^+^ **×** N_180_ attenuated salt-mediated damage in hot pepper plants. When EMs^+^ **×** N_150_ or EMs^+^ **×** N_180_ was applied, the osmoprotectants (proline and TSS), protein and capsaicin contents increased in salt-stressed hot pepper plants. In addition, the ascorbate-glutathione cycle, and the enzymatic antioxidant activity (CAT, SOD, and GR) increased. The fertilization of EMs^+^ **×** N_150_ or EMs^+^ **×** N_180_ improved the photosynthetic efficiency (SPAD chlorophyll, *Fv/Fm*, and PI), tissue water status, and MSI, which in turn, enhanced the growth and yield of hot pepper grown in saline soils. The application of EMs and/or N mediated the anatomical recovery and nutrient acquisition in hot pepper leaves. In conclusion, the application of EMs^+^ **×** N_150_ or EMs^+^ **×** N_180_ was highly effective in enhancing hot pepper growth and productivity in salt-affected soils.

## Data availability statement

The original contributions presented in the study are included in the article/supplementary material. Further inquiries can be directed to the corresponding authors.

## Author contributions

AA, TA, SA, KE and MG conceived and designed the experiments. AA, TA, IM, WS, OA, II, KH, ME, SA, KE and MG analyzed the data and drafted the manuscript. AA, TA, WS, SA, KE and MG wrote and edited the final manuscript. All authors contributed to the article and approved the submitted version.
